# Timing of Antiretroviral Therapy for HIV in the Setting of TB Treatment

**DOI:** 10.1155/2011/103917

**Published:** 2010-12-27

**Authors:** Damani A. Piggott, Petros C. Karakousis

**Affiliations:** ^1^Division of Infectious Diseases, Department of Medicine, Johns Hopkins University School of Medicine, 1550 Orleans Street, Rm 110, Baltimore, MD 21231, USA; ^2^Department of International Health, Johns Hopkins Bloomberg School of Public Health, Baltimore, MD 21205, USA

## Abstract

The convergent human immunodeficiency virus (HIV) and tuberculosis (TB) pandemics continue to collectively exact significant morbidity and mortality worldwide. Highly active antiretroviral therapy (HAART) has been a critical component in combating the scourge of these two conditions as both a preemptive and therapeutic modality. However, concomitant administration of antiretroviral and antituberculous therapies poses significant challenges, including cumulative drug toxicities, drug-drug interactions, high pill burden, and the immune reconstitution inflammatory syndrome (IRIS), thus complicating the management of coinfected individuals. This paper will review data from recent studies regarding the optimal timing of HAART initiation relative to TB treatment, with the ultimate goal of improving coinfection-related morbidity and mortality while mitigating toxicity resulting from concurrent treatment of both infections.

## 1. Convergence of HIV/TB Pandemics

There are approximately 33 million HIV-infected persons worldwide, of whom approximately 2 million are children [1]. An estimated 2 million deaths have been attributed annually to HIV/AIDS, with approximately 250,000 pediatric deaths. One third of the world's population is infected with *Mycobacterium tuberculosis*. In 2007, there were approximately 9.3 million incident cases of TB [2], with an estimated 1 million of these occurring in children [3].

The HIV pandemic has fueled a rise in both TB incidence and mortality, with an approximately 40% increase in incident TB cases compared to 20 years ago [4]. In the USA, one quarter of all TB cases occur in HIV-infected persons [5] and worldwide an estimated 1.37 million (14.8%) TB cases occur in HIV-positive persons, resulting in 456,000 TB-related deaths in this population [2]. HIV/TB coinfection is particularly prevalent in populations with limited resources. Thus, the prevalence of HIV infection among patients with TB ranges from 50% to 80% in sub-Saharan Africa, as compared to 2–15% in other parts of the world. 

HIV/TB coinfected persons have been shown to have a higher mortality rate than those without either infection alone, regardless of CD4 count [6]. TB accounts for 26% of AIDS-related deaths worldwide and 29% of TB-related mortality has been attributed to HIV infection [2]. 

An estimated 500,000 cases of multidrug-resistant (MDR) TB (defined as resistance to the first line agents rifampin and isoniazid) occur annually [7], with a similar prevalence among HIV-infected persons as for HIV-uninfected persons, as MDR-TB strains do not appear to be more transmissible or pathogenic in the setting of HIV/AIDS. However, relative to the general population, factors such as congregation, delayed diagnosis, and inadequate initial treatment, contribute to periodic outbreaks of MDR-TB in HIV-infected populations [8]. Moreover, outbreaks of extremely drug-resistant (XDR) TB (defined as MDR-TB plus resistance to any fluoroquinolone and at least one of the three injectable second-line drugs capreomycin, kanamycin, and amikacin) have been reported with increasing frequency, with particularly deadly consequences for HIV-coinfected individuals [9].

## 2. Pathogenesis of HIV/TB Coinfection

The fate of *M. tuberculosis* infection in the human host is dependent in large part on host innate and adaptive immune responses. Initial recognition of the tubercle bacillus depends upon innate immune receptor recognition of conserved pathogen-associated molecular patterns (PAMPs) of *M. tuberculosis* [10–12]. In the appropriate host cellular environment, the adaptive immune system is primed to contain, but usually not eradicate, the infection within necrotic granulomas, leading to latent TB infection. In particular, CD4 T cells are critical in the control of *M. tuberculosis *infection, as quantitative and qualitative deficiencies of these effector cells in HIV-infected individuals increase the rates of both primary and reactivation disease. While the lifetime risk of developing active TB is approximately 10% for immune-competent persons following initial infection, for persons with HIV coinfection the annual risk can exceed 10% [13], and the risk of TB reactivation rises as the CD4 cell count declines [14–16].

## 3. Prevention of Active TB in HIV-Positive Patients

Treatment of latent TB infection with isoniazid is highly effective in preventing the progression to active disease among HIV-infected persons [17, 18]. A recent meta-analysis of randomized controlled trials revealed a reduction in the incidence of active TB of over 30% in persons receiving chemoprophylactic treatment compared to those receiving placebo [19].

HAART also has been found to play an important role in preventing the development of active TB in HIV-positive patients, reducing the incidence of TB by up to 90% in patients receiving such therapy relative to those not receiving antiretroviral drugs [20–22]. However, despite viral suppression with HAART, the risk of TB remains higher in HIV-infected persons than in HIV-uninfected persons, suggesting incomplete immune restoration in the former group [23].

## 4. Potential Challenges in the Treatment of HIV-Associated TB

The concomitant treatment of HIV and active TB poses significant challenges, particularly relating to the duration and frequency of dosing of anti-TB drugs and the optimal timing of HAART initiation relative to TB treatment, which has important consequences vis-à-vis overlapping drug toxicities and drug-drug interactions between anti-TB drugs and antiretroviral drugs as well as the immune reconstitution inflammatory syndrome (IRIS). 

Although current guidelines recommend a 6-month rifamycin-based regimen for treatment of drug-susceptible pulmonary TB regardless of HIV status [24], the results of two randomized trials suggest that relapse rates after such therapy may be higher among HIV-infected persons than among HIV-uninfected persons [25, 26]. A recent meta-analysis of randomized, controlled trials and cohort studies found that at least 8 months duration of rifamycin-based therapy, daily drug dosing during the initial phase of treatment, and concurrent antiretroviral therapy are associated with improved outcomes in HIV-associated TB [27]. Although intermittent dosing under direct observation is a mainstay of TB treatment regimens in the USA and elsewhere, highly intermittent (once or twice weekly) therapy has been associated with increased relapse rates in HIV-infected persons, often with acquired rifamycin resistance [28, 29]. 

Concurrent treatment of TB and HIV is associated with a higher risk of adverse reactions compared to treatment of either infection alone. In particular, the first-line antituberculous drugs isoniazid, rifampin, and pyrazinamide may each cause hepatotoxicity, which may be compounded by concurrent use of protease inhibitors and nonnucleoside reverse transcriptase inhibitors [30, 31]. Since HIV-infected persons are at increased risk for isoniazid-induced peripheral neuropathy, these patients should take vitamin B6 and avoid antiretroviral drugs with potential peripheral neurotoxicity (e.g., stavudine and didanosine). Additionally, gastrointestinal distress and high pill burden can contribute to reduced tolerability and adherence to a combined TB/HIV therapeutic regimen. 

Pharmacokinetic interactions between HIV and TB regimens can have a significant impact on the therapeutic efficacy of each regimen. Potent induction of the cytochrome p450 system by rifampin can lead to subtherapeutic levels of the protease inhibitors accompanied by virological failure. Rifabutin, another rifamycin with less potent induction of the p450 system, may be used together with protease inhibitors, but its dose must be decreased to avoid rifabutin-related toxicity. However, rifabutin is not readily available in many resource-limited settings [32]. Rifampin also reduces levels of the nonnucleoside reverse transcriptase inhibitors efavirenz and nevirapine. Although these interactions do not appear to have a deleterious effect on virological outcomes with efavirenz-based therapy, increased rates of virological failure have been reported with concomitant nevirapine use [33, 34].

Initiation of antiretroviral therapy in HIV/TB-coinfected individuals can also result in an initial paradoxical clinical deterioration known as IRIS. IRIS has been reported in up to 30% of HIV-infected persons subsequent to HAART initiation [35–37]. Individuals with low initial CD4 counts, who experience a rapid rise in their CD4 cell count or rapid decline in their HIV viral load soon after initiating HAART, are at increased risk for developing IRIS [38–41]. TB-related IRIS may manifest as high fever, worsening pulmonary infiltrates and respiratory compromise, increasing lymphadenopathy, and neurological deterioration with the potential for severe morbidity and mortality [38–48]. 

Despite the significant challenges posed by concomitant treatment of HIV and TB infections, delaying HAART until after the completion of TB treatment in coinfected persons may carry its own attendant risks. As early antiretroviral therapy in HIV-infected persons has been shown to reduce the risk of AIDS progression and death [49], concerns have been raised that even brief delays in HAART initiation can lead to substantial increases in mortality [50]. Therefore, there has been significant interest recently in determining the optimal timing of HAART initiation relative to TB treatment in the setting of HIV-associated TB.

## 5. Optimal Timing of Antiretroviral Therapy in HIV/TB-CoInfected Persons

Leonard et al. studied HIV/TB coinfected individuals, who were followed between 1991 and 1997 at a single center in Atlanta, Georgia [51]. Increased one year survival rates were observed in the 1994 and 1997 patient cohorts compared to the 1991 cohort, suggesting a correlation between increased survival and concurrent antiretroviral and anti-TB therapy, although a direct role for HAART in improved clinical outcomes could not be inferred from this study (see Table 1). 

Three studies based in Thailand attempted to elucidate the risks and benefits of concomitant treatment of HIV and TB in coinfected individuals. A retrospective study by Sungkanuparph et al. at a single site in Thailand evaluated 29 adult patients with HIV/TB coinfection, all with CD4 counts less than 200 [52]. Antiretroviral therapy was initiated between 4 and 12 weeks after initiation of antituberculous therapy, based on clinical stability on the TB regimen. A single death in this cohort was attributed to CMV infection, and one case of IRIS was observed. Additional reported adverse events included rash with nevirapine, dizziness with efavirenz, and anemia with d4T. Although this study was limited by the relatively small sample size and lack of a control group, 26 of the 29 patients were able to complete a full course of TB treatment while taking antiretroviral drugs, suggesting the potential tolerability of dual therapy. 

Manosuthi et al. subsequently performed a larger retrospective cohort study with approximately 1000 adult HIV-infected persons with active TB diagnosed by clinical symptoms and positive sputum acid-fast smear [53]. A uniform anti-TB regimen was administered with the standard initial 2-month regimen comprising isoniazid, rifampin, pyrazinamide, and ethambutol, followed by 4 months of isoniazid and rifampin. There was some variability in the antiretroviral regimen employed with 80% of individuals receiving a nevirapine-based regimen, 16% an efavirenz-based regimen, and the remainder receiving a protease inhibitor-based regimen. Concurrent TB treatment and HAART appeared to confer a significant survival benefit, with a mortality rate of 7.7% in the group receiving both treatments, compared to 67.7% in the group receiving TB treatment alone. Although this study was limited by the greater underlying morbidity in the group not receiving HAART, with more advanced TB and higher rates of drug resistance noted in this group, subgroup analysis demonstrated significantly greater survival among patients receiving HAART within 6 months of TB diagnosis as compared to those receiving HAART beyond 6 months of TB diagnosis. However, patients in whom HAART was started within 2 months of TB treatment initiation did not appear to have improved survival relative to those who began receiving HAART 4 months after initiating TB treatment. 

A third Thai-based study by Sanguanwongse et al. also attempted to evaluate the role of HAART on survival of HIV/TB-coinfected individuals [54]. This observational cohort study evaluated 626 HIV/TB-coinfected patients receiving HAART together with TB treatment and 643 HIV/TB-coinfected patients receiving TB treatment alone. A significant decline in mortality was observed in the group receiving concurrent HAART (11%) compared to the group not receiving HAART (46%). Although this study was nonrandomized and precise information on the HAART regimen employed for each patient was lacking, it provided further support for the potential benefit of concomitant HAART and TB treatment.

Five recent studies have attempted to address the appropriate timing of initiation of HAART in HIV/TB-coinfected patients. A small retrospective study in Tehran involving 69 individuals with HIV/TB coinfection was divided into 2 groups [55]. One group, treated from 2002 to 2005, received HAART after 8 weeks of TB treatment if the CD4 count was less than 200. The second group, treated from 2005 to 2006, received HAART after 2 weeks of TB treatment if the CD4 count was less than 100 and after 8 weeks if the CD4 count was between 101 and 200. A lower mortality rate and higher rate of TB cure was observed in the latter group suggesting that early initiation of HAART may be beneficial at lower CD4 counts in HIV-associated TB. No difference in adverse events including IRIS and new opportunistic infections was reported between these 2 groups [55]. 

A partially retrospective, multicenter study in Madrid, Spain compared HAART initiation within 2 months of TB diagnosis to HAART initiation 3 months subsequent to TB diagnosis [56]. No difference in virological or immunological outcomes was observed between these 2 groups, although the early HAART group had lower mean baseline viral loads. However, early HAART initiation (within 2 months) was associated with improved survival [56]. 

The aforementioned studies were primarily observational and retrospective in nature. The need for prospective randomized controlled trials to address the clinically important question of the optimal timing of HAART initiation relative to antituberculous therapy led to the design of several such trials in recent years [60]. The Starting Antiretroviral Therapy at Three Points in Tuberculosis (SAPIT) trial, an open label, randomized controlled trial conducted in a large clinic in Durban, South Africa [58], enrolled HIV-positive adult patients with a CD4 count <500 and AFB smear-positive TB. The TB regimen consisted of a 2-month standard initial phase with rifampin, isoniazid, ethambutol, and pyrazinamide for TB treatment-naïve individuals, with the addition of streptomycin for treatment-experienced individuals, followed by rifampin and isoniazid during the continuation phase. All patients received the same HAART regimen (didanosine, lamivudine, and efavirenz) and counseling regarding medical adherence. There were 3 main arms of this study: a sequential therapy arm in which patients received HAART subsequent to completion of TB treatment, an early integrated therapy arm in which patients received HAART within 4 weeks after the start of TB treatment, and a late integrated therapy arm in which HAART was administered within 4 weeks after completion of the initial phase of treatment. Patients were randomly assigned to one of these 3 groups and the primary outcome was all-cause mortality. The sequential therapy arm was stopped early by the data and safety monitoring committee upon interim analysis of the data. A significant 56% decline in mortality was observed in the combined integrated arms compared to the sequential arm, with a mortality rate of 5.4 per 100 person-years in the former group as compared to 12.1/100 person-years in the latter group. Similar rates of virological suppression were observed in each of the groups after similar duration of HAART, and no difference was observed in grade 3 and grade 4 events between the groups.

The initial findings of the SAPIT trial provide compelling evidence for the superiority of integrated versus sequential antituberculous and antiretroviral therapy for coinfected individuals. Further elucidation of the precise timing of HAART initiation relative to TB treatment has been provided recently by the Cambodian Early versus Late Introduction of Antiretrovirals (CAMELIA) trial [59]. This study was an open label, prospective, randomized controlled trial enrolling HIV-positive adult patients with a CD4 count <200/mm^3^ and AFB smear-positive TB at 5 sites in rural and urban Cambodia. Patients were treated with a 2-month intensive phase TB regimen consisting of rifampin, isoniazid, pyrazinamide, and ethambutol, followed by a 4 month continuation phase regimen of rifampin and isoniazid. Participants were randomized to 2 arms, an early arm in which HAART was introduced 2 weeks subsequent to the initiation of antituberculous therapy and a late arm in which HAART was introduced 8 weeks subsequent to the initiation of antituberculous therapy. Patients in each arm received a HAART regimen consisting primarily of lamivudine, stavudine, and efavirenz. The preliminary report from this study noted a significant 34% decline in mortality in the early HAART arm compared to the late HAART arm, with a mortality rate of 8.28 per 100 person-years in the early arm as compared to 13.77 per 100 person-years in the late arm. Similar rates of virological suppression were observed in both groups.

The AIDS Clinical Trials Group recently completed a study entitled “A Strategy Study of Immediate Versus Deferred Initiation of Antiretroviral Therapy for HIV Infected Persons Treated for Tuberculosis With CD4 Less Than 200 Cells/mm^3^” [61]. This study was an open-label, randomized controlled trial conducted between August 2006 and July 2010, which enrolled persons aged >13 years old with CD4 count less than 200 cells/mm^3^ and confirmed or probable TB. Participants were assigned to early initiation of HAART within 2 weeks after initiating TB treatment, or deferral of HAART until 8 to 12 weeks after initiation of TB treatment. The majority of participants received efavirenz, tenofovir, and emtricitabine and the primary outcome measure was the proportion of participants who have survived without AIDS progression. The analysis of this study is pending.

## 6. Conclusions and Future Directions

The findings of multiple retrospective studies and of the prospective randomized SAPIT and trial provide compelling evidence that initiation of HAART should not be delayed pending completion of TB treatment for HIV/TB-coinfected individuals. The most recent WHO guidelines for antiretroviral therapy in adolescents and adults recommend the initiation of HAART between 2 and 8 weeks subsequent to the initiation of TB therapy for severely immunosuppressed coinfected individuals, as defined by a CD4 count <200 mm^3^ [62]. The preliminary reported findings from the CAMELIA trial provide evidence that coinfected individuals with CD4 counts <200 mm^3^ would benefit from HAART initiation early during this intensive phase of TB treatment, that is at 2 weeks as opposed to deferral of HAART to 8 weeks after-initiation of TB therapy [59]. Further multicountry data on the timing of HAART for coinfected individuals with CD4 counts <200 mm^3^ is likely to be provided by the recently concluded ACTG A5221 study [61]. 

An increased risk of IRIS was observed in the integrated arm of the SAPIT trial (12% in the integrated arm versus 3.8% in sequential arm) as well as in the early arm of the CAMELIA trial (4.03 per 100 person months in the early arm versus 1.44 per 100 person months in the late arm), consistent with the findings of previous studies regarding the development of IRIS relative to HAART initiation [63–66]. However, the lack of mortality or changes in antiretroviral regimen attributable to IRIS in the SAPIT trial, coupled with the findings of a prior modeling study demonstrating the benefit of early HAART initiation in the setting of low mortality rates from TB-IRIS [67], provides further support for early HAART initiation in persons with HIV-associated TB. Although guidelines have been developed specifically for resource-limited settings [68], the ability to detect and appropriately treat IRIS in a timely fashion outside a controlled study environment requires further study. 

The risk of IRIS may vary depending on the type of opportunistic pathogen in a site-specific manner. This concept may be of particular clinical relevance for central nervous system (CNS) infections. CNS-IRIS occurs after HAART initiation in approximately 1% of cases [69]. Immune restoration in the setting of CNS opportunistic infections can lead to increased intracranial pressure as a result of the inflammatory response in a relatively closed space, with potentially irreversible neurologic sequelae [70, 71]. High mortality rates have been reported in cases of CNS IRIS, including up to 57% for such pathogens as *Cryptococcus neoformans* [72, 73]. A recent study showed increased mortality with early initiation of HAART in the setting of concomitant HIV/cryptococcal meningitis [74]. Extrapulmonary TB, including CNS disease (manifesting as tuberculous meningitis or CNS tuberculoma), may demonstrate a different clinical course relative to primary pulmonary disease post-HAART initiation [43, 45, 74–77]. It should be noted that the great majority of patients included in the SAPIT trial had exclusively pulmonary TB, not allowing conclusions to be drawn regarding the optimal timing of HAART initiation in the setting of CNS and other forms of extrapulmonary TB. Whereas a greater proportion of participants in the CAMELIA trial had extrapulmonary TB, it is not certain whether subgroup analysis by location of involvement could be performed. However, preliminary reports of a single center randomized trial in Vietnam examining HAART initiation in patients with HIV and CNS-TB suggested no mortality difference in the group treated immediately with HAART (within 7 days of TB treatment) as compared to the group in whom HAART was deferred (2 months post-randomization) [57]. However, there were higher rates of grade 4 adverse events in the immediate HAART arm, suggesting that deferral of HAART initiation to the continuation phase of TB treatment may be warranted in the setting of HIV and CNS-TB coinfection. The optimal timing of HAART initiation for individuals with extrapulmonary TB, as well as for those with other opportunistic infections, requires further study.

As for adults, HIV is a major risk factor for the development of pediatric TB [78]. Recommendations for deferral of HAART until after completion of TB treatment for children with mild or insignificant immunodeficiency had initially been proposed. The most recent WHO guidelines recommend initiation of HAART as soon as tuberculosis therapy is tolerated, ideally as early as 2 weeks and no later than 8 weeks subsequent to initiation of TB treatment, regardless of clinical stage and degree of immunosuppression [79]. However, studies on the optimal timing of HAART initiation among children with TB have been limited to observational reports, although a recent such study suggested a trend towards decreased mortality and higher rates of virological suppression with earlier HAART initiation in this age group [80]. Extrapolation of findings from adult studies to the pediatric population may not be possible given potential differences in pharmacokinetics and pharmacodynamics as well as challenges in definitive pediatric TB diagnosis. Further data is required to evaluate current recommendations for this specific population. 

HIV patients with drug-resistant TB demonstrate reduced survival compared to those with drug-susceptible TB [9, 53, 81]. Many additional challenges exist in the comanagement of MDR-TB and XDR-TB among HIV-positive populations, including delays in the diagnosis of drug resistance, and inadequate infection control modalities, as well as the decreased efficacy, prolonged duration, and increased cost of therapeutic regimens compared to those for drug-susceptible TB [82]. Moreover, second- and third-line TB regimens demonstrate their own distinct cumulative toxicities with concomitant HAART administration. For instance, the nephrotoxicity associated with tenofovir may be compounded by the antituberculous aminoglycosides and the peripheral neurotoxicity induced by stavudine and didanosine and psychiatric disturbances associated with efavirenz may be exacerbated by the antituberculous agent cycloserine. Additionally, the pill burden and gastrointestinal distress associated with drug-susceptible TB regimens are even greater with MDR-TB and XDR-TB regimens [82, 83]. The timing of HAART initiation in the setting of drug-resistant TB thus requires careful consideration of both the unique cumulative toxicities as well as the distinctive pharmacokinetic interactions between antiretroviral drugs and second- and third-line TB regimens [84]. Further investigation is warranted to determine if the findings of the SAPIT and CAMELIA studies are applicable to persons coinfected with HIV and drug-resistant TB. Moreover, the anticipated emergence of HIV resistance in resource-limited settings will further complicate the management of HIV/TB coinfected patients. 

Several studies have shown a benefit to initiating HAART at higher CD4 counts [85–87]. As a consequence, WHO has recently increased the recommended CD4 threshold for HAART initiation from 200 to 350/mm^3^ for stage 1 or 2 disease [62]. HAART initiation at even higher CD4 counts has been recommended by other groups [88]. Whereas tuberculosis is a recommended indication for initiation of HAART at any CD4 count, the WHO guidelines state that “there are limited data on the initiation of ART in patients with TB and CD4 counts of >350 cells/mm^3^” [62]. The SAPIT trial suggested a trend towards a mortality benefit when HAART was initiated in persons with CD4 counts of 200–500/mm^3^ in the integrated groups compared to the sequential group though the sample size in this subgroup was small. Although the CAMELIA trial addresses the management of HIV/TB coinfected persons with CD4 counts <200/mm^3^, recommendations regarding the optimal timing of HAART for coinfected individuals with CD4 counts >200/mm^3^ may not be derived from this trial or from the recently concluded ACTG A5221 study. Such a determination, with significant implications for the management of HIV/TB coinfection worldwide, awaits future study, including the results of the comparative analysis of the early and late integrated arms of the SAPIT trial. 

Only approximately 42% of those in need worldwide currently receive HAART [89]. The appropriate treatment of HIV/TB-coinfected individuals has the potential to significantly reduce morbidity and mortality globally, however the ability to deliver such treatment remains a critical challenge. The available data should provide an impetus for improving access to both life-saving HIV and TB medications to all those in need of such therapy. 

## Figures and Tables

**Table 1:** Studies on initiation of highly active antiretroviral therapy (HAART) in HIV/TB-coinfected individuals.

Study (author, year)	Study characteristics	Timing of HAART relative to TB treatment	Outcomes	Adverse events
Leonard et al. 2002 [51]	- Retrospective cohort - 644 adults and children (60% pulmonary TB, 25% pulmonary and extrapulmonary TB, 15% extrapulmonary TB) - Single center — Atlanta, GA - Comparison of 3 HIV/TB coinfected cohorts: 1991, 1994, 1997	HAART at time of diagnosis versus HAART post-TB diagnosis — not defined	Decreased 1 year mortality in 1994 and 1997 cohort compared to 1991 cohort	N/A

Sungkanuparph et al. 2006 [52]	- Retrospective, observational - 29 adult patients (69% pulmonary TB, 31% extrapulmonary TB) - Jan 2002–Dec 2002 - Single center—Thailand	Median 8 weeks (range 4–12 weeks)	Virological suppression -65% at 24 weeks -76% at 48 weeks TB outcomes -26/29 completed therapy -No relapse -No new OI	IRIS—1/29 Death—1/29 (CMV encephalitis) Rash—2/29 (NVP) EFV switched to NVP 1/29 (dizziness) AZT switched to D4T 3/29 (anemia)

Manosuthi et al. 2006 [53]	- Retrospective cohort - 1003 adult patients - Jan 2000–Dec 2004 - Single center — Thailand - Comparison of HAART+ to HAART− group	2 mth versus 4 mth versus 6 mth versus 9 mth versus 12 mth (subgroup analysis)	Increased survival for pts. receiving HAART as compared to no HAART Increased mortality with HAART initiation at >6 mths. (11.3%) compared to <6 mths (4.1%) (*P* = .018)	N/A

Sanguanwongse et al. 2008 [54]	- Observational cohort - 1269 patients (adult and pediatric) (54% pulmonary TB, 35% extrapulmonary TB, 12% both) - Oct 2004–March 2006 - Multicenter — Thailand National Surveillance Network	Not defined	Decreased mortality in group receiving HAART (11%) compared to that not receiving HAART (46%) (relative risk 0.24, 95% confidence interval: 0.19 to 0.30)	N/A

Tabarsi et al. 2009 [55]	- Retrospective cohort - 69 patients - 2002–2006	Group I: HAART after 8 wks if CD4 < 100 Group II: HAART at 2 wks if CD4 < 100 Group I & II: HAART after 8 wk if CD4 101–200	Increased mortality when HAART deferred after 8 weeks if CD4 <100 Group I versus Group II mortality = 27.7% versus 4.5% ( *P* = .03)	No difference between groups I and II regarding Grade 3 or 4 events, IRIS, or new OI

Velasco et al. 2009 [56]	- Mixed retrospective/ prospective study - 313 patients - 1996–2004	- Simultaneous: HAART within 2 mths of TB diagnosis - Nonsimultaneous: HAART after 3 mths of TB diagnosis	- Decreased mortality in simultaneous group (9.3%) versus nonsimultaneous group (19.7%) (*P* = .011) - No difference in virological/immunological outcomes	N/A

Torok et al. 2009 [57]	- Randomized, double blind, placebo-controlled trial - 253 patients - Vietnam - Clinical diagnosis of TB meningitis	HAART started within 7 days (immediate arm) or at 2 mths (deferred arm) after initiation of TB treatment	Mortality: -Immediate arm: 76 deaths/127 pts -Deferred arm: 70 deaths/126 pts (HR = 1.16, *P* = .31)	Incidence of grade 3 or 4 adverse events first 2 mths: -Immediate: 86% -Deferred: 75% (*P* = .04)

Abdool Karim et al. 2010 [58]	- Open-label, randomized, controlled trial - 642 patients - Durban, South Africa - Only patients with positive sputum smear for acid-fast bacilli and CD4 count < 500/mm^3^ included	HAART started either during TB treatment (in two integrated-therapy groups) or after completion of TB treatment (in one sequential-therapy group)	Mortality rate per 100 py: -Integrated arm: 5.4 -Sequential arm: 12.1 (*P* = .003) Virological suppression 6 mths after HAART initiation: -Integrated arm: 91.1% -Sequential arm: 86.7% (*P* = .4)	Incidence of IRIS: -Integrated arm: 12.4% -Sequential arm: 3.8% (*P* ≤ .001) Grade 3 or 4 adverse events: Integrated arm: 30/100 py Sequential arm: 32/100 py (*P* = .69)

Blanc et al. 2010 [59]	- Open label randomized, controlled trial - 661 patients - Cambodia - Only patients with positive smear for acid-fast bacilli and CD4 count < 200/mm^3^ included	HAART started at 2 weeks (early arm) or 8 weeks (late arm) after initiation of TB treatment	Mortality rate per 100 py: -Early arm: 8.28 -Late arm: 13.77 (*P* = .002) Virological suppression 50 wks after HAART initiation: -Early arm: 95.6% -Late arm: 95.6% (*P* = .82)	Incidence of IRIS per 100 pm: -Early arm: 4.03 -Late arm: 1.44 (*P* < .0001)

OI: Opportunistic infections IRIS: Immune reconstitution inflammatory syndrome EFV: Efavirenz NVP: Nevirapine pts: Patients HR: Hazard ratio py: Person-years pm: Person-months N/A: Not available.
